# Supplementary material for: Targeted Treatment of Sarcomas by Single Protein Encapsulated Doxorubicin with Undetectable Cardiotoxicity and Superior Efficacy
Source: Cancers (Basel). 2025 Mar 4;17(5):881. doi: 10.3390/cancers17050881 (PMC11899045; doi:10.3390/cancers17050881)
Supplement: Supplementary file 1 [file cancers-17-00881-s001.zip › cancers-3465854-supplementary.pdf]

Supplementary Information

Targeted Treatment of Sarcomas by Single Protein Encapsulated Doxorubicin with Undetectable Cardiotoxicity and Superior Efficacy

Changjun Yu[1,2*], Faqing Huang[3*], Leslie Wang[2], Mengmeng Liu[2], Warren A. Chow[4], Xiang Ling[5,6], Fengzhi Li[5], Galen Cook-Wiens[7], Linrong Li[8,9] and Xiaojiang Cui[9*]

Author Affiliations:

[1]Division of Chemistry and Chemical Engineering, California Institute of Technology, 1200 E California Blvd, Pasadena, CA 91125, USA

[2]Sunstate Biosciences LLC, 118 S. Berkeley Ave, Pasadena, CA 91107, USA; leslie.wang@sunstatebiosciences.com (L.W.); mml@sunstatebiosciences.com (M.L.)

[3]Department of Chemistry and Biochemistry, The University of Southern Mississippi, Hattiesburg, MS 39406, USA

[4]Division of Hematology and Oncology, Department of Medicine, UCI Health, Orange, CA 92868, USA; wachow@hs.uci.edu

[5]Department of Pharmacology and Therapeutics, Roswell Park Comprehensive Cancer Center, Elm and Carlton Streets, Buffalo, NY 14263, USA; xiang.ling@roswellpark.org (X.L.); fengzhi.li@roswellpark.org (F.L.)

[6]Canget BioTekpharma LLC, 701 Ellicott Street, Buffalo, NY 14203, USA

[7]Department of Biomedical Sciences, Cedars Sinai Medical Center, Los Angeles, CA 90048, USA; galen.cook-wiens@cshs.org

[8]Department of Breast Surgery, Peking Union Medical College Hospital, Chinese Academy of Medical Sciences and Peking Union Medical College, Beijing 100006, China; lilinrong2018@126.com

[9]Department of Surgery, Samuel Oschin Comprehensive Cancer Institute, Cedars Sinai Medical Center, Los Angeles, CA 90048, USA

* Correspondence: cjyu@caltech.edu (C.Y.); faqing.huang@usm.edu (F.H.); xiaojiang.cui@cshs.org (X.C.)

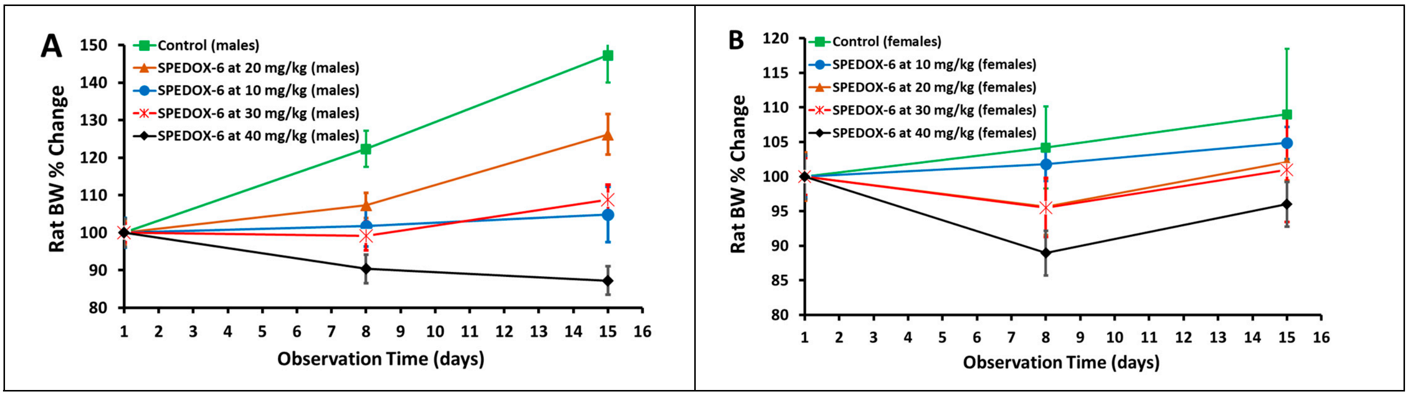

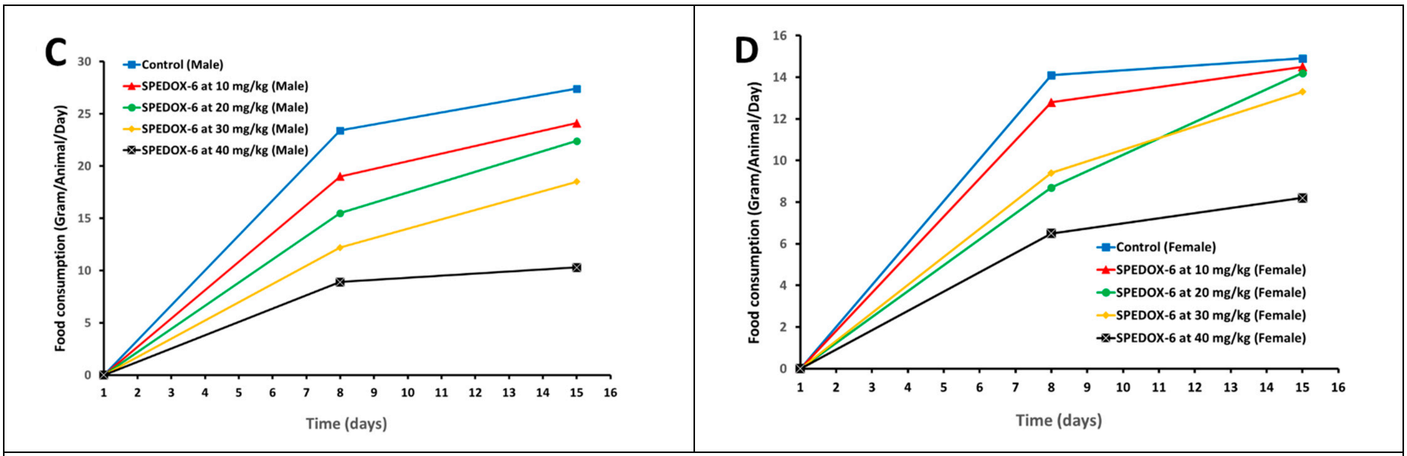

Figure S1. SD rat BW change and food consumption at different doses of non-GLP grade SPEDOX-6. A) Male rat BW change; B) Female rat BW change, C) Male rat food consumption; D) Female rat food consumption.

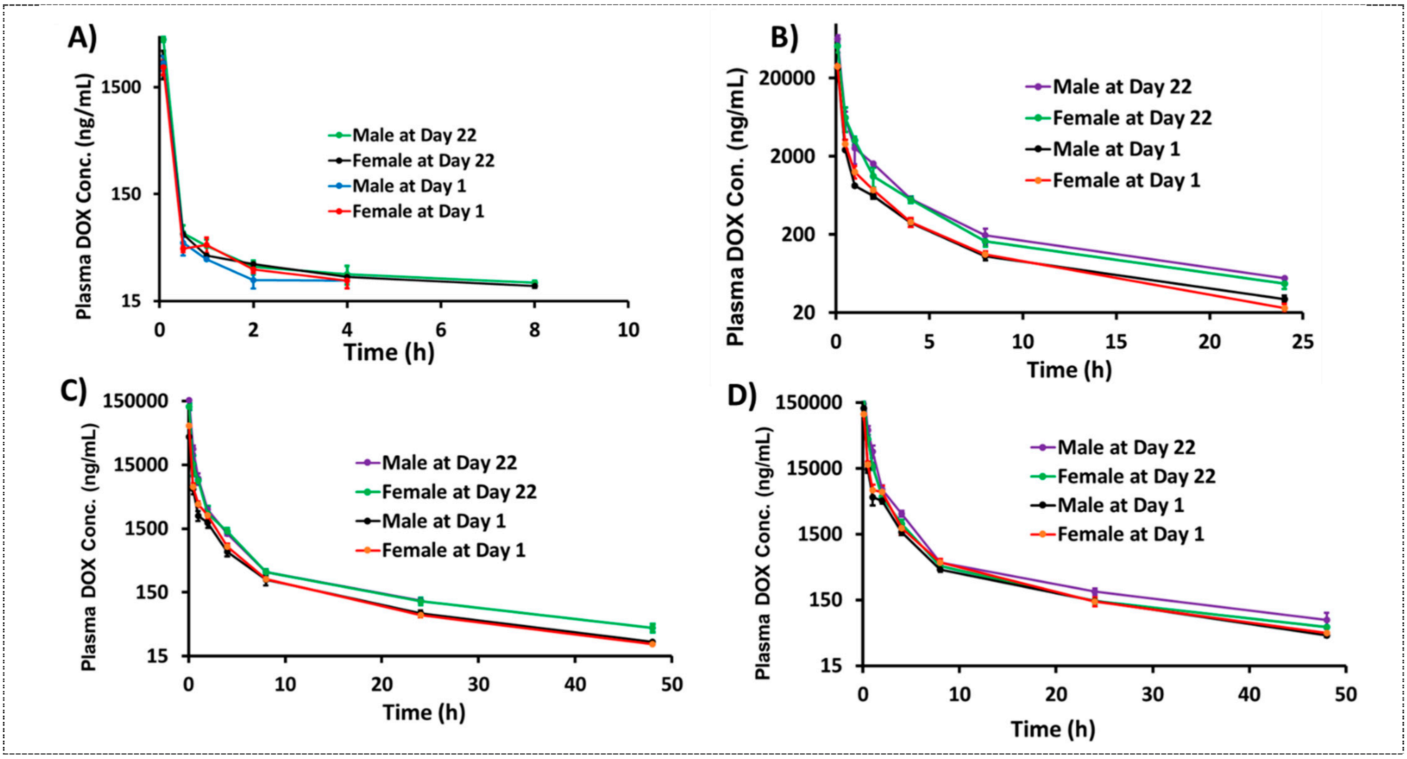

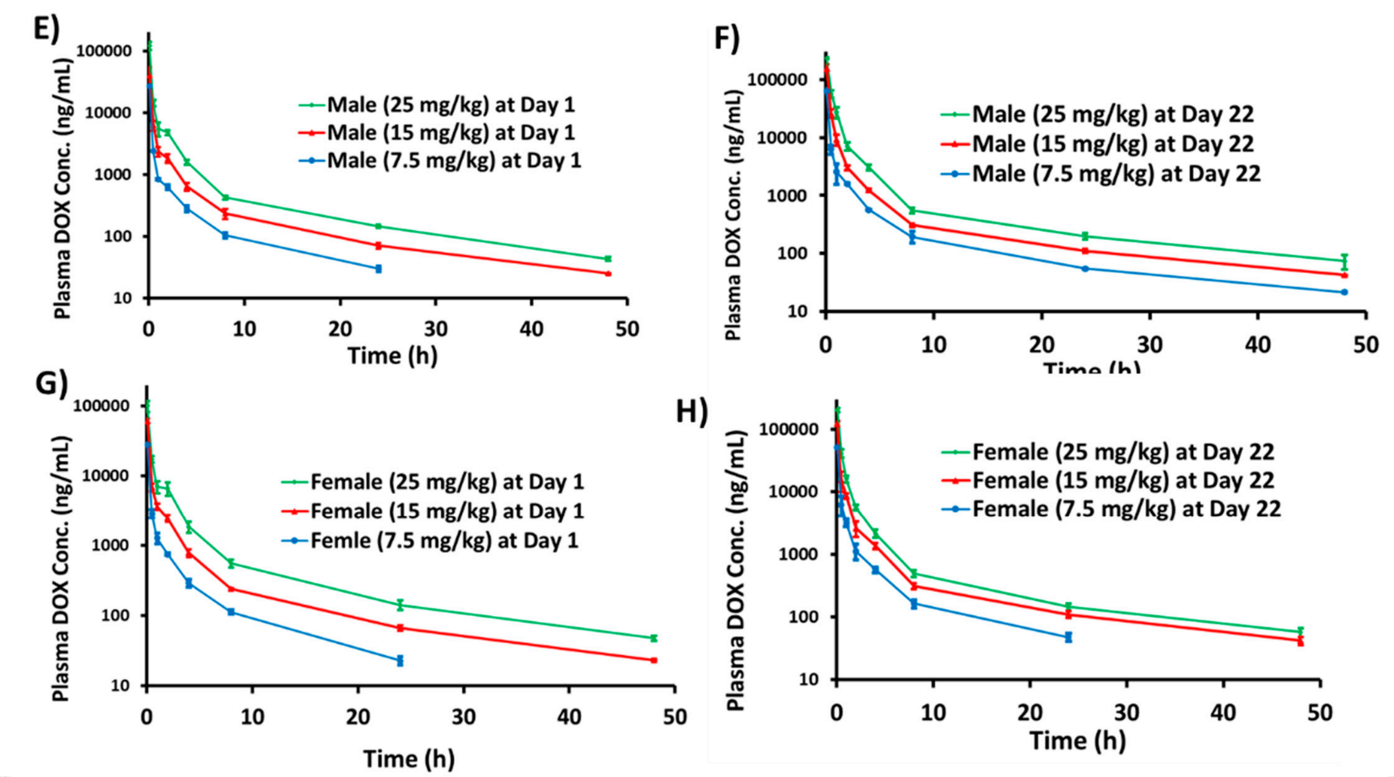

Figure S2. SD rat serum DOX concentration-time profiles of different doses of DOX and GLP-grade SPEDOX-6. A) Dox at 3.5 mg/kg for males and females on Day 1 and Day 22; B) SPEDOX-6 at 7.5 mg/kg for males and females on Day 1 and Day 22; C) SPEDOX-6 at 15 mg/kg for males and females on Day 1 and Day 22; D) SPEDOX-6 at 7.5 mg/kg for males and females on Day 1 and Day 22; E) Male rats at doses of 7.5, 15 and 25 mg/kg of SPEDOX-6 on Day 1; F) Male rats at doses of 7.5, 15 and 25 mg/kg of SPEDOX-6 on Day 22; G) Female rats at doses of 7.5, 15 and 25 mg/kg of SPEDOX-6 on Day 1, (H) Female rats at doses of 7.5, 15 and 25 mg/kg of SPEDOX-6 on Day 22.

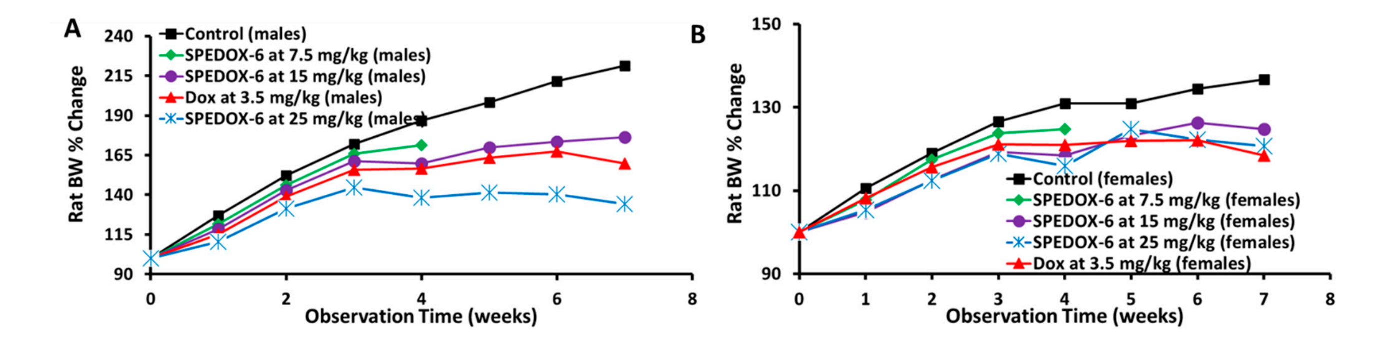

Figure S3. SD rat BW % change at different doses of DOX and GLP-grade SPEDOX-6. A) male rats BW % change; B) female rats BW % change.

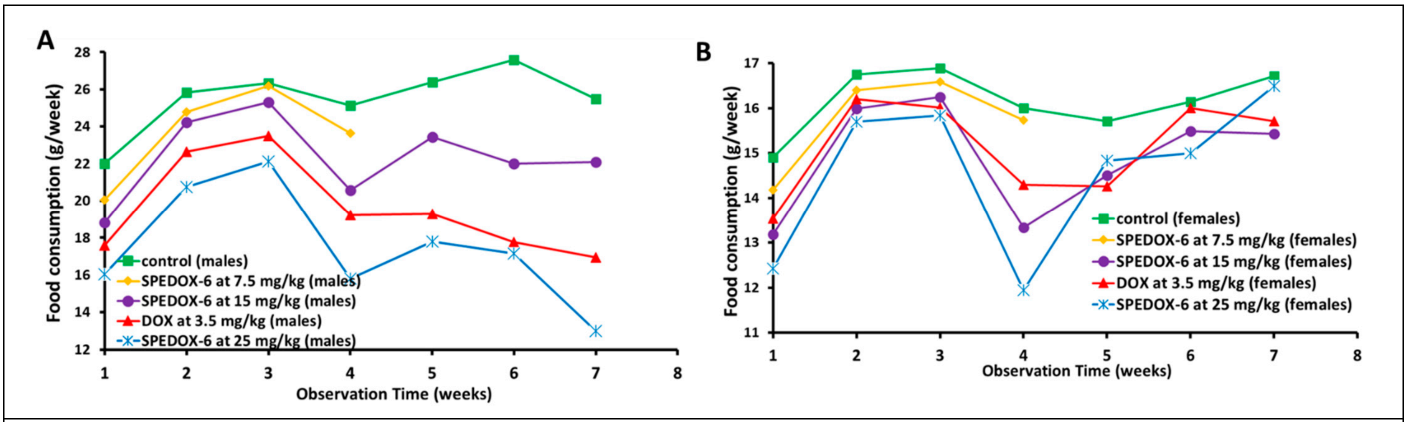

Figure S4. SD rats food consumption at different doses of DOX and GLP-grade SPEDOX-6. A) male rat food consumption; B) female rat food consumption.

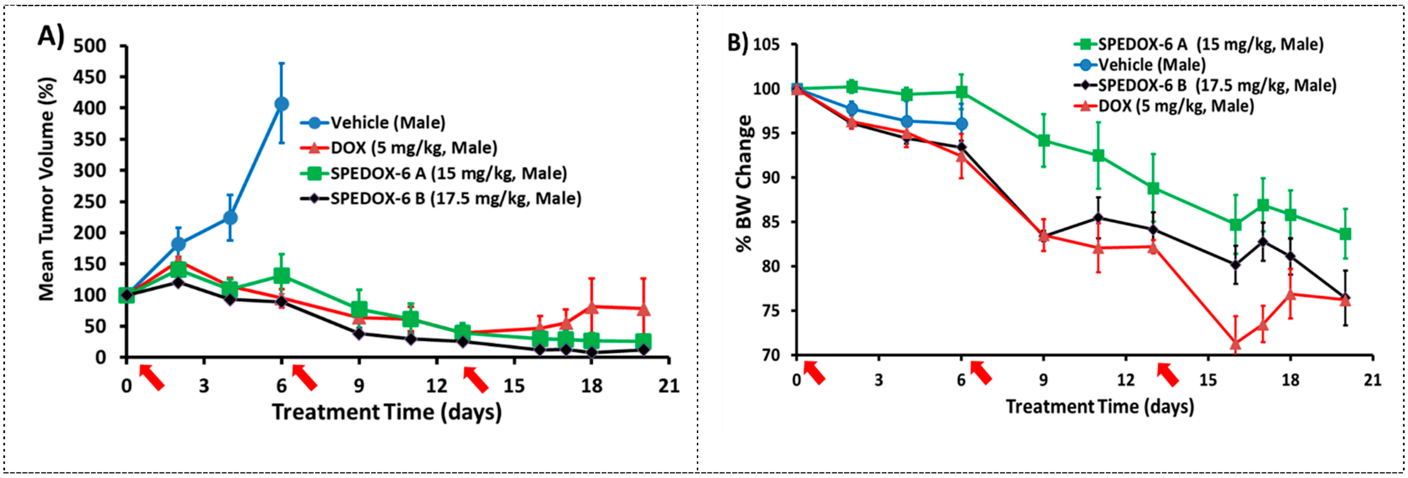

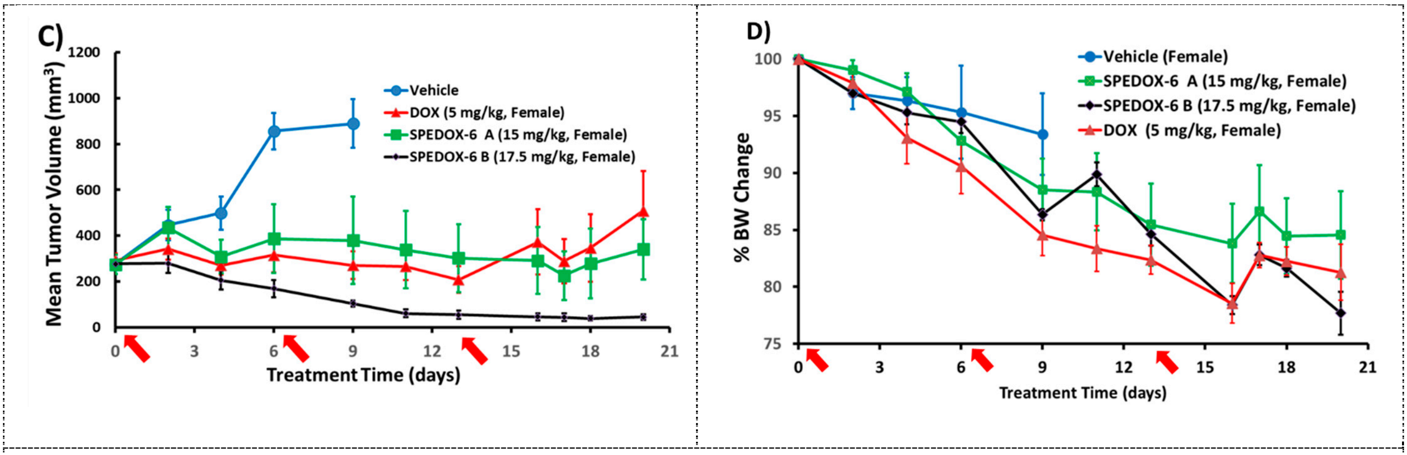

Figure S5. Antitumor efficacy and toxicity of SPEDOX-6 for male and female mice, drug injections at Day 0, 6 and 13, indicated by arrow signs; A) Mean TV vs treatment time for male mice. Mice # for each group, control (n = 4), DOX treatment (n= 4), SPEDOX-6 A at 15 mg/kg (n= 5) and SPEDOX-6 B at 17.5 mg/kg (n= 5), In DOX treatment group, one mouse had to be sacrificed at Day 12 due to severe BW loss; B) Mean BW change vs treatment time for male mice, no significant difference was observed among the four groups C) Mean TV vs treatment time for female mice. Mice # for each group, control (n = 4), DOX treatment (n= 4), SPEDOX-6 A at 15 mg/kg (n= 4) and SPEDOX-6 B at 17.5 mg/kg (n= 5). On Day 20, SPEDOX-6 B group was significantly different from DOX group (p = 0.0359); D) Mean BW change vs treatment time for female mice, no significant difference was obseved among the four groups**.**

GLP-Toxicology Study of SPEDOX-6:

Non-GLP dosing range study of SPEDOX-6:

During the study, neither mortality nor moribundity was noted in animals in negative control group, 10, 20 and 30 mg/kg groups. One female animal (#2150618) in the 40 mg/kg group was found dead on Day 12. Hunched back, thin, decreased spontaneous motor, hair fluffy of whole body, red/brown oral discharge and head scab were observed before the animal died. Macroscopically, small thymus and thin body were noted. Microscopically, severe decreased cellularity of lymphocyte in cortex and medulla was noted in the thymus. The animal death was noted in the high dose group, which was considered to be test article-related, although the

incidence of death was low. It is speculated that the cause of death was considered to be possibly related to the test article due to lack of systemic histopathological examination.

Some abnormal clinical signs, including thin, hunched back, decreased spontaneous motor, pale, hair fluffy and oral discharge were noted in animals from the 20, 30 and 40 mg/kg groups, which were considered to be test article related due to the dose- and time-response relationship. In addition, sparse hair (2/5), alopecia (2/5), eye discharge (1/5) and loose stools (1/5) in males and scab (2/5) in females were noted in the 40 mg/kg group. The changes in clinical observations could not be judged to be test article-related, since they were only noted in the 40 mg/kg group, suggesting that more attention should be paid in the future study.

Decreases in body weights, body weight gains or food consumptions were noted in animals in the 10, 20, 30 and 40 mg/kg groups as compared to the concurrent negative control group in the same sex. These changes were considered to be test article-related due to the dose- and/or time-response relationship and obvious magnitude. 1 week after dosing, except for male animals in the 40 mg/kg group, a tendency to recover of body weight gains and food consumption was noted in other animals. At terminal sacrifice (Day 15), decreases in the organ weight, organ-to-body/brain weight ratio of the thymus and adrenal glands in animals, decreases in the organ weight, organ-to-body/brain weight ratio of the heart in females, and decreases in the organ weight, organ-to-body/brain weight ratio of the testes, prostate and seminal vesicles in males were noted in the 10, 20, 30 and 40 mg/kg groups as compared to the concurrent negative control in the same sex. These changes were considered to be test article-related due to obvious magnitude, or related changes were noted in the microscopic observations.

In addition, as compared to the concurrent negative control group in the same sex, statistically significant changes in organ weight, organ-to-brain/ body weight ratio were noted in animals in the 20, 30 and 40 mg/kg groups. For 20 mg/kg group, there are increases in the organ-to-body weight ratio of the brain and lungs with bronchi in males. For 30 mg/kg group, there are decreases in the organ weight of the lungs with bronchi, increases in the organ-to-body weight ratio of the brain, lungs with bronchi, liver and kidneys in males, and increases in the organ-to-body weight ratio of the liver in females. For 40 mg/kg group, there are decreases in the organ weight of the brain, lungs with bronchi and epididymides, increases in the organ-to-body weight ratio of the brain, lungs with bronchi, liver and kidneys and decrease in the organ-to-brain weight ratio of the epididymides in males, and increases in the organ weight, organ-to- brain/body weight ratio of the liver, increases in the organ-to- brain/body weight ratio of the kidneys and increase in the organ- to- body weight ratio of the spleen in females. The changes were possibly related to be test article due to obvious magnitude, suggesting that more attention should be paid in future study.

Test article-related microscopic findings were identified at the thymus, spleen, prostate gland and seminal vesicles of animals in the 20, 30 and 40 mg/kg groups at scheduled necropsy on Day 15, including: moderate to severe decreased cellularity of lymphocyte in cortex and medulla of the thymus (20 mg/kg: 2/5

males, 3/5 females; 30 mg/kg: 4/5 males, 3/5 females; 40 mg/kg: 5/5 males, 4/5 females); minimal to slight vacuolation of macrophage in the thymus (30 mg/kg: 1/5 male; 40 mg/kg: 5/5 males); slight decreased cellularity of lymphocyte in white pulp of the spleen (40 mg/kg: 1/5 male); minimal to moderate diffuse atrophy of the prostate gland and seminal vesicles (40 mg/kg: 4/5 males). They were considered to be test article-related since the lesions were of high incidence, and with the dose-response relationship, and was not noted in the negative control group.

GLP-Toxicology Study of SPEDOX-6:

Objective: The objectives of this study were to evaluate toxicity, toxic target organs and toxicokinetics (TK) profile of SPEDOX-6 administered by intravenous infusion to Sprague-Dawley (SD) rats once every 3 weeks for 4 consecutive weeks (2 doses in total), and to evaluate the reversibility of toxicity following a 3-Week Recovery period. A comparative study was carried out with the positive control article (Doxorubicin Hydrochloride for Injection) to provide animal study data for the follow-up study of the test article.

Method: Based on the body weight, a total of 214 SD rats (107 rats/sex) were randomly assigned to 10 groups with 15/sex/group in Groups 1, 2, 4 and 5 for toxicity study, 10 rats/sex/group in Groups 3 for toxicity study, 5 rats /sex in Group 6 for toxicokinetic study, and 8 rats /sex/group in Groups 7, 8, 9 and 10 for toxicokinetic study. The rats in Groups 1 and 6 were administered with Sodium Chloride Injection as the negative control groups (0 mg/kg); the rats in Groups 2 and 7 were administered with Doxorubicin Hydrochloride for Injection as the positive control groups (calculated by DOX, 3.5 mg/kg); the rats in Groups 3 and 8, Groups 4 and 9, and Groups 5 and 10 were administered with 7.5, 15, and 25 mg/kg of SPEDOX-6 (DOX equivalent, always defined as DOX equivalent for SPEDOX-6 in this entire report) as the low, middle and high dose groups, respectively. The animals were administered by intravenous infusion via tail vein once every three weeks for 4 consecutive weeks, and 2 doses in total, and had a 3-week recovery period. The dose volume was 10 mL/kg and the dosing speed was set at 3.33 mL/kg/min. The first dosing day was defined as Day 1.

Parameters evaluated in this study included those of clinical observations (including injection site observation), body weight, food consumption, ophthalmoscopic examinations, hematology, coagulation, clinical chemistry, urinalysis and toxicokinetics (TK). The first 10 rats/sex/group in Groups 1 to 5 were euthanized one week after the last dosing (on Day 29), and the remaining 5 rats/sex/group were euthanized on Day 50 following a 3-week recovery period. All animals in Groups 1 to 5 were subjected to a complete necropsy examination, organ weighing, and macroscopic examination. Histopathological evaluation was performed on the animals in Groups 1 and 5.

Results:

The formulations of SPEDOX-6 at the concentrations of 0.75, 1.5 or 2.5 mg/mL and the positive control article (DOX) at the concentration of 0.35 mg/mL were analyzed for concentration and homogeneity after the

preparation on Day 1 and Day 22. The actual concentrations of SPEDOX- 6 in the test article formulations ranged from 106.16% to 109.28% of the nominal concentrations, and the concentration coefficient of variation (CV%) of the upper, middle, and lower layers of each formulation ranged from 0.00% to 0.86%, the actual concentrations of positive control article formulations ranged from 93.99% to 95.58% of the nominal concentrations, and the concentration coefficient of variation (CV%) of the upper, middle, and lower layers of each formulation ranged from 0.24% to 0.64%,indicating that the formulations were properly prepared and acceptable for use. Neither mortality nor moribundity was noted in animals in each group throughout the study.

25 mg/kg dose group: Neither mortality nor moribundity was noted in animals in this group. The test article-related changes observed in this dose group mainly included: Hunched back (3/15 males and 3/15 females) and abnormal appearance (dark red urine, 1/15 males and 3/15 females) were noted throughout the study. Decreased body weight and food consumption were noted in animals from Week 1. At the end of dosing (Day 29), decreased White Blood Cell-related parameters (WBC, Neut, Lymph, Mono, Eos and Baso), Red Blood Cell-related parameters (RBC, HGB and Retic) and PLT in hematology were noted in animals. Shortened PT (only in males) and APTT and increased FIB in coagulation parameters were noted in animals. Decreased TP, Alb and A/G, increased GGT (only in males), UREA (only in males), CHO and TG in clinical chemistry; increases in the positive rates of BLD, PRO and LEU in urine analysis in animals. Decreased organ weights of thymus, spleen and adrenal gland, increased organ weights of liver were noted in animals, and decreased organ weights of testes, prostate gland and seminal vesicles were noted in male animals. Increased erythrocyte series, decreased lymphocyte series and granulocyte/erythrocyte, increased erythroid hyperplasia in bone marrow smear were noted in animals. In macroscopic observation, small thymus with an incidence of 13/20 were observed. In microscopic observation, decreased cellularity and increased adipocytes in bone marrow (femur & sternum), fibrosis in femoral bone marrow, hemorrhage in sternal bone marrow; decreased lymphocytic cellularity in thymus, spleen and lymph node (mesenteric, mandibular), hemorrhage in medulla in mesenteric lymph node; hyaline cast, degeneration/ regeneration in proximal tubule and vacuolation in glomerulus in kidneys; glycogen accumulation of hepatocytes in liver; atrophy of lobule of mammary glands in male animals; degeneration in sciatic nerve; germ cell depletion of seminiferous tubule in testes; atrophy of ventral lobe and/or dorsal lobe in prostate gland; atrophy and epithelial single cell necrosis in seminal vesicles; secretory depletion of acinar cell in parotid gland, lacrimal glands and pancreas were observed. After the 3-week recovery period (Day 50), partial recovery tendencies were observed in food consumption, hematology, bone marrow smear and organ weight. In macroscopic observation, small tests were observed. The lesions in thymus and lymph nodes (mesenteric and mandibular) were not present. Other abnormal parameters did not show recovery tendencies. No test article-related abnormal changes in ophthalmoscopic examinations and no test article-related cardiovascular toxicity were observed in animals in this dose group.

15 mg/kg dose group: Neither mortality nor moribundity was noted in animals in this group. The change of each parameter was similar to that of the 25 mg/kg dose group, while the change degrees were comparable with or less than the 25 mg/kg dose group, mainly included: abnormal appearance (dark red urine), decreased body weight and food consumption. At the end of dosing (Day 29), decreased White Blood Cell-related parameters (WBC, Neut, Lymph, Mono, Eos and Baso), Red Blood Cell-related parameters (RBC and Retic) and PLT in hematology were noted in animals. Shortened PT (only in males) and APTT and increased FIB in coagulation parameters were noted in animals. Decreased TP and Alb, increased GGT and CHO in clinical chemistry were noted in animals. Increases in the positive rates of BLD, PRO and LEU in urine analysis were observed in animals. Decreased organ weights of thymus and spleen were observed in animals, decreased organ weights of testes, prostate gland and seminal vesicles were also observed in male animals, and decreased organ weights of adrenal gland were also observed in female animals. Increased erythrocyte series, decreased lymphocyte series and granulocyte/erythrocyte, increased erythroid hyperplasia in bone marrow smear were observed. In macroscopic observation, small thymus with an incidence of 5/20 were observed. In microscopic observation, decreased cellularity and increased adipocytes in bone marrow (femur & sternum); decreased lymphocytic cellularity in thymus, spleen and lymph node (mesenteric, mandibular), hemorrhage in medulla in mesenteric lymph node; hyaline cast in proximal tubule and vacuolation in glomerulus in kidneys; atrophy of lobule of mammary glands in male animals; degeneration in sciatic nerve; germ cell depletion of seminiferous tubule in testes; atrophy of ventral lobe and/or dorsal lobe in prostate gland; atrophy and epithelial single cell necrosis in seminal vesicles; secretory depletion of acinar cell in parotid gland, lacrimal glands and pancreas were observed, which the change degrees and severity were comparable with or less than the 25 mg/kg dose group. After the 3-week recovery period (Day 50), partial recovery tendencies were observed in food consumption, hematology, clinical chemistry and organ weight. In macroscopic observation, small tests were observed. The lesions in thymus and lymph nodes (mesenteric and mandibular) were not present. Other abnormal parameters did not show significant recovery tendencies. No test article-related abnormal changes in ophthalmoscopic examinations and no test article-related cardiovascular toxicity were observed in animals in this dose group.

7.5 mg/kg dose group: Neither mortality nor moribundity was noted in animals in this group. The change or change degrees of each parameter was less than the 15 mg/kg dose group, mainly included: Minimal decreased body weight and food consumption were observed in animals. At the end of dosing (Day 29), minimal decreased White Blood Cell-related parameters (WBC, Neut, Lymph), and increased Red Blood Cell-related parameters (Retic) in hematology were noted in animals. Decreased Alb (only in males) and increased GGT (only in males) in clinical chemistry were noted in animals. Increases in the positive rates of BLD and LEU in urine analysis were observed in animals. Decreased organ weights of spleen were observed in male animals, and decreased organ weights of thymus were observed in female animals. Decreased lymphocyte series

in bone marrow smear were observed. In microscopic observation, decreased cellularity in femoral bone marrow, increased adipocytes in bone marrow (femoral and sternal); decreased lymphocytic cellularity in marginal zone of spleen; atrophy of lobule of mammary glands in male animals were observed. The extent and incidence of lesions were less than or equivalent to the 15 mg/kg dose group of the same sex. No test article-related abnormal changes in clinical observation, ophthalmoscopic examinations, coagulation and macroscopic observation were observed in animals in this dose group.

Positive control group (3.5 mg/kg DOX): Neither mortality nor moribundity was noted in animals in this group. The change of each parameter was similar to that of the 25 mg/kg dose group, mainly included: Hunched back and decreased body weight and food consumption were observed in animals throughout the study. At the end of dosing (Day 29), decreased White Blood Cell-related parameters (WBC, Neut, Lymph, Mono, Eos and Baso), Red Blood Cell-related parameters (RBC, HGB and Retic) and PLT in hematology were noted in animals. Shortened PT and APTT and increased FIB in coagulation parameters were noted in animals. Decreased TP, Alb and A/G, increased GGT (only in males), CHO and TG in clinical chemistry were noted in animals. Increases in the positive rates of BLD, PRO and LEU in urine analysis were observed in animals, which the change degrees were comparable with or less than the 25 mg/kg dose group. Decreased organ weights of thymus and spleen and increased organ weights of liver in animals, and decreased organ weights of testes, epididymides and seminal vesicles in males, and decreased organ weights of adrenal gland in females were observed. Increased erythrocyte series, decreased lymphocyte series and granulocyte/erythrocyte, increased erythroid hyperplasia in bone marrow smear were observed. In macroscopic observation, small thymus with an incidence of 5/20 were observed. In microscopic observation, decreased cellularity and increased adipocytes in bone marrow (femur & sternum); decreased lymphocytic cellularity in thymus, spleen and lymph node (mesenteric, mandibular), hemorrhage in medulla in mesenteric lymph node; hyaline cast, degeneration/regeneration in proximal tubule and vacuolation in glomerulus in kidneys; atrophy of lobule of mammary glands in male animals; degeneration in sciatic nerve; germ cell depletion of seminiferous tubule in testes; atrophy of ventral lobe and/or dorsal lobe in prostate gland; atrophy and epithelial single cell necrosis in seminal vesicles; secretory depletion of acinar cell in parotid gland, lacrimal glands and pancreas were observed. After the 3-week recovery period (Day 50), partial recovery tendencies were observed in food consumption, hematology and organ weight. In macroscopic observation, small tests were observed. Other abnormal parameters did not show significant recovery tendencies. No positive control article-related abnormal changes in ophthalmoscopic examinations and the cardiovascular toxicity were observed in animals in this dose group.

Statistical and graphical analyses

Part 1: HT-1080 mouse model study

Overall statistical analysis: A linear mixed model was used for tumor volume at Day 6 and Day 20, controlling for day zero tumor volume, group, day, and a group by day interaction and gender. A similar model was used for body weight percentage but did not control for day zero since the percentage is calculated from day zero. A compound symmetric covariance was used to model the correlation in repeated measures. Post-hoc pairwise comparisons between groups at each day were made using a Tukey-Kramer adjustment for multiple testing. DF is the degrees of the freedom.

DOX at 5 mg/kg is designated as DOX group, SPEDOX-6 at 15 mg/kg and 17.5 mg/kg are designated as SPEDOX-6 A and SPEDOX-6 B, respectively. **Table S1** shows t test pair-wise comparisons between groups and times with the post hoc Tukey-Kramer adjustment to the *p*-values. These tests are averaged over gender. On Day 6 the control group was very significantly different from the other three groups (**, p <0.0001, n = 8, n = 9 and n = 10 for all three comparisons). On Day 6 the control group was very significantly different from the other three groups (**, p <0.0001, n = 8, n = 9 and n = 10, for all three comparisons). On day 6 the DOX treatment group did not show a significant difference from SPEDOX-6 A (ns, p = 0.8682, n = 9) or SPEDOX-6 B (ns, p = 0.9688, n = 10), nor did the two SPEDOX-6 groups show a significant difference from each other (ns, p = 0.3098, n = 10). On Day 20 the DOX treatment group did not show a significant difference from SPEDOX-6 A group (ns, p = 0.3264, n = 9), but did show a significantly higher average tumor volume compared to the SPEDOX-6 B group (*, p = 0.0175, n = 10). The two SPEDOX-6 groups did not show a significant difference from each other on day 20 (ns, p = 0.7345, n = 10).

Tumor volume statistical analysis in mice by gender at day 20: A regression model was fit with tumor volume at day 20 as the outcome, adjusting for baseline tumor volume, and with group and gender as covariates. The differences in means may be large but there are large standard deviations and few mice. The interaction between group and gender was not significant (type III F test P = 0.2337, n = 5), implying that while the average difference may be different between genders and groups, gender did not significantly alter the differences between groups at Day 20.

The model for BW percentage of baseline showed significant effects for group and day differences, but neither for the interaction of group by day nor for gender. After adjustment for multiple testing the group differences were not significant at Day 6 or Day 20. There were significant differences over time in the three drug groups (**Table S2**). On Day 6 the control group did not show significantly higher average BW percentage compared to DOX group (ns, p = 0.6719, n = 8), SPEDOX-6 A group (ns, p = 0.9998, n = 9), or SPEDOX-6 B group (ns, p = 0.9900, n = 10). Average BW percentage was lower but not significant in the DOX group compared to SPEDOX-6 A (ns, p = 0.4254, n = 9) or SPEDOX-6 B (ns, p = 0.9515, n = 10). SPEDOX-6 A group's average BW percentage was slightly higher than SPEDOX-6 B group's but not significant (ns, p = 0.9144, n = 10). On Day 20 SPEDOX-6 B group had lower BW percentage than DOX group, but not significant (ns, p = 0.9769, n = 8), with a similar result for SPEDOX-6 B and SPEDOX-6 A (ns, p = 0.0879, n = 9). On day

20 SPEDOX-6 B1was also not significantly higher in average BW percentage compared to DOX (ns, p = 0.5252, n = 8). There were very significant decreases in BW percentage from Day 6 to Day 20 within DOX (**, p = 0.0003, n = 8), and both SPEDOX-6 B and SPEDOX-6 A (**, $p < 0.0001$, n = 10 and n = 9 for both).

A model using the outcome of Day 20 tumor volume adjusting for baseline tumor volume was made to test differences among the groups in only male mice and the post-hoc pairwise differences used a Tukey-Kramer adjustment to the p-values (**Table S3**). The only significant difference between groups among only male mice was between DOX group and SPEDOX-6 B group, before adjustment (* p = 0.0493, n = 5) but after adjustment for multiplicity this was not significant (ns, adjusted p = 0.1118, n = 5).

The same model was used for female mice on Day 20 (**Table S4**). The only significant difference between groups among only female mice was between DOX group and SPEDOX-6 B before adjustment, and after adjustment for multiplicity this was still significant (adjusted p = 0.0359, n = 5).

Table S1: **Summary of Tumor Volume Changes for All Groups**

Group	day	vs Group	vs day	Average tumor volume diff. Estimate	Standard Error	DF	Adjusted *p*-value
DOX	6	SPEDOX-6 A	6	-114.90	92.0857	51	0.8682
DOX	6	SPEDOX-6 B	6	80.7521	89.5908	51	0.9688
DOX	6	Control	6	-674.39	94.4842	51	**<.0001****
DOX	6	DOX	20	-84.1863	93.0601	25.4	0.9683
SPEDOX-6 A	6	SPEDOX-6 B	6	195.65	87.1780	50.9	0.3098
SPEDOX-6 A	6	Control	6	-559.49	92.3752	50.9	**<.0001****
SPEDOX-6 A	6	SPEDOX-6 A	20	242.26	84.0629	23.7	0.0987
SPEDOX-6 B	6	Control	6	-755.14	89.6167	51	**<.0001****
SPEDOX-6 B	6	SPEDOX-6 B	20	178.55	79.7491	23.7	0.3124
DOX	20	SPEDOX-6 A	20	211.54	95.7106	51.2	0.3264
DOX	20	SPEDOX-6 B	20	343.48	93.5010	51.3	**0.0175***
SPEDOX-6 A	20	SPEDOX-6 B	20	131.94	87.1780	50.9	0.7345

.

Table S2. Summary of BW Change (%) for All Groups

Group	day	vs group	vs day	Average BW% difference estimate	Standard error	DF	Adjusted *p*-value
DOX	6	SPEDOX-6 A	6	-5.0737	2.5032	48.9	0.4254
DOX	6	SPEDOX-6 B	6	-2.4151	2.4422	48.9	0.9515
DOX	6	Control	6	-4.1691	2.5743	48.9	0.6719
DOX	6	DOX	20	12.3150	2.2886	24.2	0.0003**

Table S2. Summary of BW Change (%) for All Groups							
**Group**	**day**	**vs group**	**vs day**	**Average BW% difference estimate**	**Standard error**	**DF**	**Adjusted *p*-value**
SPEDOX-6 A	6	SPEDOX-6 B	6	2.6586	2.3671	48.9	0.9144
SPEDOX-6 A	6	Control	6	0.9046	2.5032	48.9	0.9998
SPEDOX-6 A	6	SPEDOX-6 A	20	12.5578	2.0565	22.7	<.0001**
SPEDOX-6 B	6	Control	6	-1.7540	2.4422	48.9	0.9900
SPEDOX-6 B	6	SPEDOX-6 B	20	16.8805	1.9510	22.7	<.0001**
DOX	20	SPEDOX-6 A	20	-4.8309	2.5998	50	0.5252
DOX	20	SPEDOX-6 B	20	2.1504	2.5386	50	0.9769
SPEDOX-6 A	20	SPEDOX-6 B	20	6.9813	2.3671	48.9	0.0879

Table S3. Differences of Least Squares Means for Male Mice								
Group	Group	Estimate	Standard error	DF	t Value	*p*-value for t	Adjustment	Adjusted *p*-value
DOX	SPEDOX-6 A	183.31	97.8236	9	1.87	0.0937	Tukey-Kramer	0.2015
DOX	SPEDOX-6 B	219.88	96.8070	9	2.27	0.0493*	Tukey-Kramer	0.1118
SPEDOX-6 A	SPEDOX-6 B	36.57	85.8328	9	0.43	0.6800	Tukey-Kramer	0.9058

Table S4. Differences of Least Squares Means for Female Mice								
Group	Group	Estimate	Standard error	DF	t Value	*p*-value for t	Adjustment	Adjusted *p*-value
DOX	SPEDOX-6 A	133.32	152.26	9	0.88	0.4040	Tukey-Kramer	0.6681
DOX	SPEDOX-6 B	433.57	144.29	9	3.00	0.0148*	Tukey-Kramer	**0.0359***
SPEDOX-6 A	SPEDOX-6 B	300.25	143.43	9	2.09	0.0658	Tukey-Kramer	0.1462

Table S5. TV of Mice Treated with 15 mg/kg SPEDOX-6 on Different Days*									
Day	Male					Female			
	M1	M2	M3	M4	M5	M1	M2	M3	M4
0	255.6	196.7	362.6	351.4	557.1	356.0	158.8	303.0	282.0
2	461.3	196.8	347.9	549.1	943.5	602.8	197.2	544.6	397.7
4	362.9	158.9	212.4	462.4	742.7	480.6	126.1	335.2	291.6
6	324.0	164.6	111.1	750.0	1110.1	823.3	142.9	307.2	273.6
9	245.8	0	46.6	435.0	865.8	912.4	18.7	367.5	221.1
11	215.3	0	18.7	377.2	619.2	806.7	18.4	323.7	207.0
13	142.0	0	8.5	222.3	416.6	713.5	15.9	270.4	207.0
16	128.6	0	0	148.7	314.6	702.5	20.4	188.2	253.7
17	191.7	0	0	79.9	257.9	525.7	23.9	193.4	160.9
18	139.6	0	0	107.9	280.9	714.2	20.8	126.7	251.4
20	152.1	0	0	105.8	203.2	647.5	49.7	214.2	453.9

Table S6. TV of Mice Treated with 17.5 mg/kg SPEDOX-6 on Different Days*										
Day	Male					Female				
	M1	M2	M3	M4	M5	M1	M2	M3	M4	M5
0	318.8	317.1	233.3	351.7	285.7	232.6	305.2	235.5	325.3	286.9
2	351.4	685.9	244.4	258.9	277.8	276.1	248.9	156.8	421.7	295.5
4	277.4	426. 5	177.4	242.8	282.1	143.8	240.5	88.2	325.3	228.7
6	277.4	410.7	192.5	185.0	272.8	109.4	189.3	95.2	306.3	145.2
9	89.1	176.4	142.8	52.7	88.8	80.6	121.0	84.6	152.1	79.5
11	84.9	167.7	109.3	10.2	53.7	52.7	47.4	30.2	129.0	45.5
13	68.8	142.9	93.1	0	57.2	48.0	47.6	18.9	125.6	33.8
16	25.6	67.7	45.8	0	31.3	94.5	37.8	14.2	71.2	14.1
17	35.5	75.9	34.1	0	36.8	57.6	20.6	17.6	105.0	16.3
18	27.0	30.7	37.2	0	17.9	56.4	29.8	27.1	66.40	13.1
20	24.8	69.3	43.9	0	33.5	62.4	57.6	22.5	73.20	11.1

*TV unit, mm^3

– partial remission

– complete remission
